# Triple adenocarcinoma pulmonar primario sincrónico: Un reto diagnóstico

**DOI:** 10.23938/ASSN.1104

**Published:** 2025-04-25

**Authors:** Pablo Andrés Ordóñez Lozano, Marco Patricio Bravo Mendoza, Andrea Carilla Sanromán

**Affiliations:** 1 Servicio Aragonés de Salud Hospital Universitario Miguel Servet Servicio de Cirugía Torácica Zaragoza España; 2 Servicio Aragonés de Salud Hospital Universitario Miguel Servet Servicio de Anatomía Patológica Zaragoza España

**Keywords:** Neoplasias Primarias Múltiples, Carcinoma de Pulmón de Células no Pequeñas, Secuenciación de Nucleótidos de Alto Rendimiento, Cirugía Torácica Asistida por Video, Neoplasms, Multiple Primary, Carcinoma, Non-Small-Cell Lung, High-Throughput Nucleotide Sequencing, Thoracic Surgery Video-Assisted

## Abstract

El cáncer de pulmón primario múltiple puede ser sincrónico o metacrónico, según el momento de aparición de la lesión. A pesar de los criterios de clasificación del cáncer de pulmón con afectación pulmonar múltiple, a veces es un reto distinguir entre el cáncer de pulmón primario múltiple y metástasis intrapulmonar. Además, hay que resaltar la importancia en diferenciar entre un cáncer de pulmón primario múltiple sincrónico y un cáncer de pulmón multifocal en vidrio deslustrado/lepídico. Aunque las características histológicas son útiles en algunos casos, a menudo se necesita un análisis molecular.

Se presenta el caso de una paciente con tres lesiones pulmonares que fue diagnosticada de triple carcinoma pulmonar primario sincrónico, con lesiones del mismo tipo histológico pero con diferentes patrones histológicos y características moleculares. En casos de cáncer de pulmón con afectación pulmonar múltiple es fundamental realizar un estudio anatomopatológico detallado para obtener un diagnóstico preciso que permita al paciente recibir el tratamiento indicado.

## Abstract (en)

Multiple primary lung cancer can be synchronous or metachronous based on the timing of lesion onset. Despite the existing classification criteria for lung cancer with multiple lung involvement, it is sometimes challenging to distinguish between lung cancer with multiple lung involvement and intrapulmonary metastases. Moreover, differentiating synchronous multiple primary lung cancer from multifocal ground-glass/lepidic-pattern lung cancer is crucial. While histologic evaluation can aid in diagnosis, molecular analyses are often necessary.

We present the case of a female patient diagnosed with triple synchronous primary lung adenocarcinomas. Although the lesions shared the same histological subtype, they exhibited distinct histological patterns and molecular profiles. In cases of lung cancer with multiple lung involvement, it is essential to perform a detailed pathological study for an accurate diagnosis and appropriate therapeutic interventions.

## INTRODUCCIÓN

El cáncer de pulmón primario múltiple (CPPM), hace referencia a la presencia de dos o más tumores de orígenes independientes en el pulmón, que pueden ser sincrónicos o metacrónicos según el momento de aparición de la lesión. En ocasiones, y a pesar de los criterios de clasificación del cáncer de pulmón con afectación pulmonar múltiple^1^, es un reto distinguir entre CPPM sincrónico y metástasis intrapulmonar^2^. Es fundamental comprender la relación que tienen las distintas lesiones entre sí para tomar las decisiones correctas sobre el tratamiento, ya que esta información distingue las lesiones tumorales independientes en estadios iniciales, del cáncer de pulmón localmente avanzado o cáncer metastásico^3^.

A continuación, se presenta el caso de una paciente con tres lesiones pulmonares tumorales para ilustrar el detallado proceso que condujo al diagnóstico de triple carcinoma pulmonar primario sincrónico.

## CASO CLÍNICO

Mujer de 65 años, con antecedente de melanoma en espalda intervenido hace más de 25 años y exfumadora desde hace más de 20 años. Acudió al sistema sanitario por pérdida ponderal.

**Figura 1:**
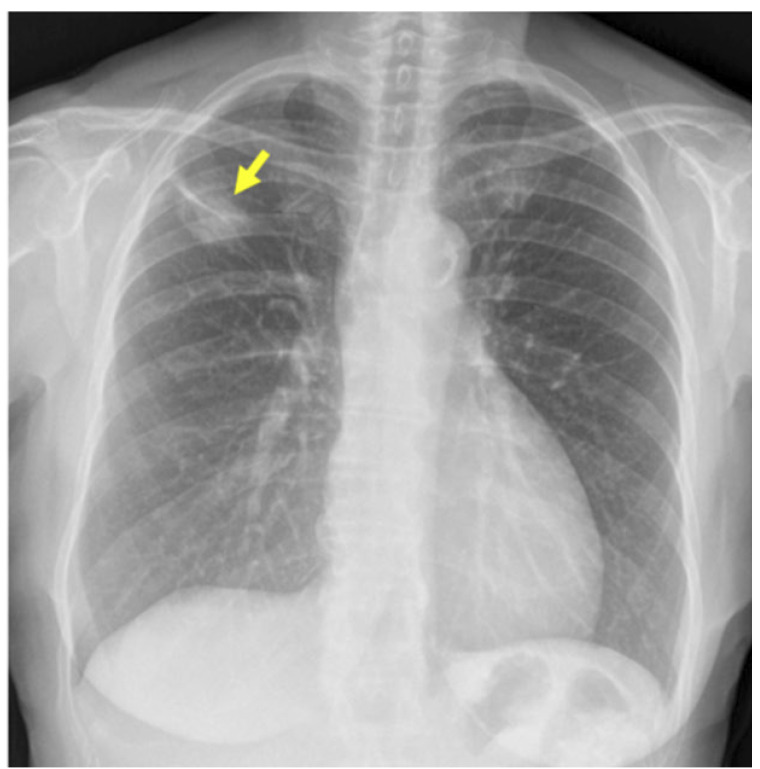
Radiografía de tórax preoperatoria. Muestra opacidad pseudonodular de 25 mm a nivel del LSD, en relación a tracto fibroso (flecha amarilla).

Se le realizó una radiografía de tórax, detectándose una opacidad pseudonodular de 25 mm en el lóbulo pulmonar superior derecho (LSD) (Fig. 1).

**Figura 2:**
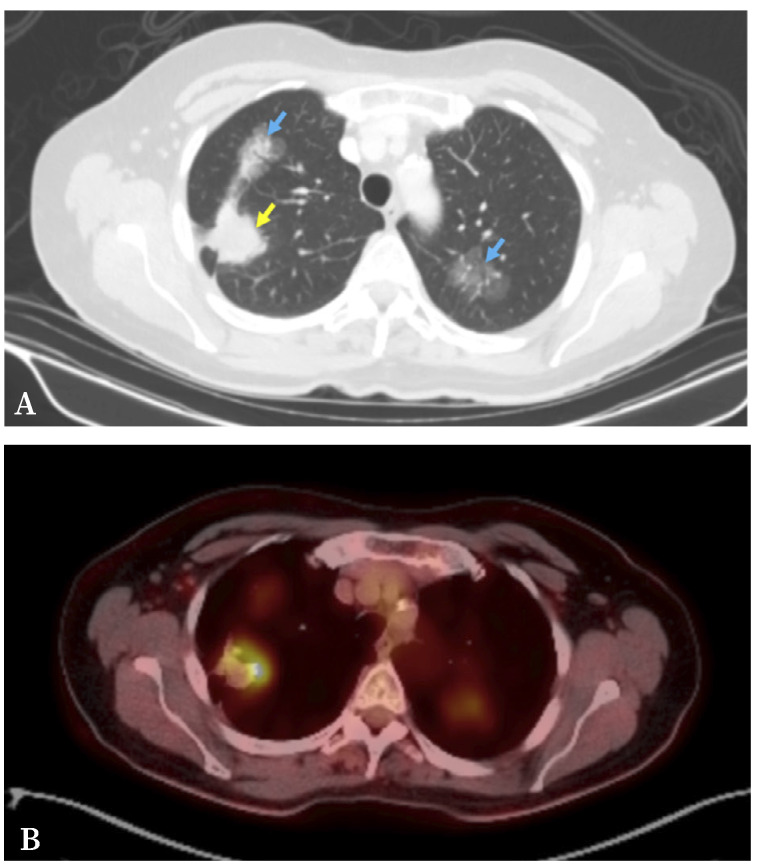
A. Corte axial de tomografía computarizada. Se observa una masa pulmonar en el lóbulo superior derecho (LSD_1_) de contornos espiculados de 37 x 30 mm con cola pleural sugestiva de probable carcinoma pulmonar (flecha amarilla). Nódulo parcialmente sólido en el lóbulo superior derecho con signo de broncograma aéreo (LSD_2_) y nódulo en vidrio deslustrado en el lóbulo superior izquierdo (flechas azules). B. Corte axial de la tomografía por emisión de positrones/tomografía computarizada que muestra la captación de los nódulos pulmonares.

Debido a este hallazgo, se amplió el estudio con una tomografía computarizada (TC) de tórax, en la que se observó una masa pulmonar en el LSD de contornos espiculados de 37 x 30 mm con cola pleural sugestiva de probable carcinoma pulmonar (LSD_1_). Además, se observaron dos nódulos subsólidos en ambos lóbulos superiores derecho (LSD_2_) e izquierdo (LSI) de posible origen inflamatorio residual, sin poder descartar malignidad (Fig. 2A).

A continuación se realizó una tomografía por emisión de positrones/TC (PET/TC) con flúor-18-fluorodesoxiglucosa, que detectó que la masa pulmonar LSD_1_ mostraba aumento del metabolismo glucosídico con un valor de captación máximo estandarizado (SUV_max_) de 8,66. Los SUV_max_ del nódulo subsólido LSD_2_ y del nódulo LSI (1,62 y 3,03) fueron compatibles con lesiones infecciosas/inflamatorias, sin poder descartar malignidad. No se observaron adenopatías hipermetabólicas regionales ni metástasis a distancia (Fig. 2B).

Durante la broncoscopia no se observaron lesiones endobronquiales. Los resultados microbiológicos del lavado broncoalveolar fueron negativos, sin signos de malignidad en la citología, igual que para el cepillado bronquial. La biopsia transbronquial derecha se diagnosticó de adenocarcinoma pulmonar primario. Durante la ecobroncoscopia (EBUS) no se observaron signos ecográficos de malignidad en los ganglios linfáticos hiliares y mediastínicos, por lo que no se tomaron muestras. Se realizó una biopsia con aguja gruesa (BAG) de la lesión en el LSI, con diagnóstico de adenocarcinoma pulmonar primario. La espirometría y la capacidad de difusión del monóxido de carbono fueron normales.

Dado los hallazgos, se planificaron dos intervenciones quirúrgicas de manera bilateral, secuencial y diferida.

En un primer momento, se realizó lobectomía superior derecha y linfadenectomía mediante cirugía torácica video-asistida (VATS) biportal, sin incidencias. Durante su estancia hospitalaria, la paciente mostró una adecuada evolución postoperatoria; presentó una fuga aérea persistente que se resolvió en el séptimo día postoperatorio.

**Figura 3:**
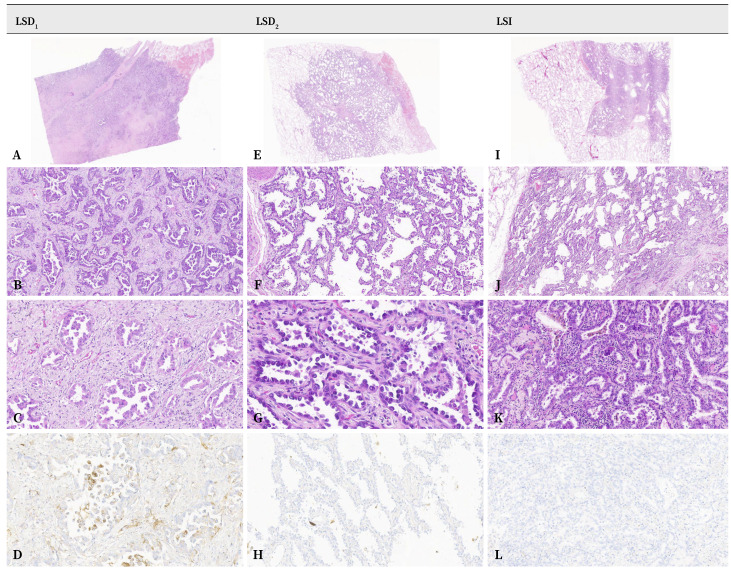
Microscopía óptica. Estudio histológico con hematoxilina-eosina (HE) e inmunohistoquímico para PD-L1 de las tres lesiones resecadas. **LSD**_1_: lesión en lóbulo pulmonar superior derecho. Lesión pulmonar intraparenquimatosa sólida (**A**), diagnosticada de adenocarcinoma pulmonar, muestra un patrón predominantemente acinar (**B**) y en menor medida papilar (**C**). Se observó positividad para PD-L1 en el 20% de las células tumorales (**D**). **LSD**_2_: lesión en lóbulo pulmonar superior derecho. Parénquima pulmonar en el que se observa una lesión tumoral sólida y bien delimitada, de localización subpleural (**E**), diagnosticada de adenocarcinoma pulmonar de patrón predominantemente lepidico (**F**, detalle en **G**). No se observaron células tumorales inmunorreactivas frente a PD-L1 (**H**). **LSI**: Lesión en lóbulo superior izquierdo. Parénquima pulmonar en el que se identifica una neoplasia sólida, de bordes espiculados y localización intraparenquimatosa (**I**), diagnosticada de adenocarcinoma pulmonar de patrones lepídico (**J**) y acinar (**K**). No se observaron células tumorales inmunorreactivas frente a PD-L1 (**L**).

El estudio anatomopatológico identificó las dos lesiones de la pieza quirúrgica obtenida en esta primera cirugía como dos tumores primarios unifocales: un adenocarcinoma invasivo, con extensa fibrosis central pT2a/N0/M0 (estadio I B), positivo para PD-L1 y con *KRAS* mutado, y un segundo tumor pulmonar primario compatible con adenocarcinoma invasivo, predominantemente lepídico, pT1b/N0/M0 (estadio I A2), con *EGFR* mutado, estadificados según la 8ª edición de la clasificación TNM (tumor, ganglio linfático y metástasis) del *American Joint Committee on Cancer* (AJCC) (Fig. 3, Tabla 1).

A los dos meses de la primera cirugía se realizó trisegmentectomía apical del LSI y linfadenectomía mediante VATS biportal, sin incidencias. Durante su estancia hospitalaria, la paciente mostró una adecuada evolución postoperatoria. El séptimo día postoperatorio se le realizó una hemopleurodesis con sangre autóloga ante la presencia de fuga aérea persistente; tras su resolución se retiró el drenaje torácico a las 48 horas y la paciente fue dada de alta hospitalaria.

El diagnóstico anatomopatológico de la lesión obtenida en la segunda cirugía correspondió a un adenocarcinoma invasivo pT1b/N0/M0 (estadio I A2), según la 8ª edición de la clasificación TNM del AJCC (Fig. 3, Tabla 1).

A, E, I: 3x; B, F: 50x; C, D, H, K, L: 100x, G: 200x; J: 20x.

Tras el alta hospitalaria de la segunda cirugía, la paciente requirió reingreso hospitalario por presentar enfisema subcutáneo secundario a episodios de tos. Días después, durante el ingreso, se colocó un drenaje torácico debido a la presencia de hidroneumotórax izquierdo, que posteriormente se resolvió.

Los resultados de los estudios moleculares, tanto dirigidos como de la secuenciación de nueva generación (NGS), apoyaron la naturaleza primaria de cada uno de los tres tumores. Se presentó el caso en un comité multidisciplinar y, tras la valoración por Oncología Médica, se consideró que la paciente no precisaba tratamiento adyuvante.

**Tabla 1:** Características anatomopatológicas de los tumores sincrónicos

Tumor	Tamaño*	Tipo histológico	Estadio	Biomarcadores
		Patrones histológicos	TNM	
		Otros patrones		
LSD_1_	39 mm	Adenocarcinoma invasivo	pT2a	*PD-L1:*
		70% acinar, 20% papilar, 5% lepídico, 5% micropapilar	N0	- positivo (20% de las células tumorales)
		Moderadamente diferenciado.	M0	*Genes mutados:*
		STAS: no se observa	I B	*- KRAS* en el codón 12
		Sin invasión de pleura visceral (PL0)		*Genes no mutados:*
		Sin invasión linfovascular		*- EGFR, ALK*, R*OS1*, *NRAS, BRAF*
LSD_2_	16 mm	Adenocarcinoma invasivo	pT1b	*PD-L1:*
		50% lepídico, 40% papilar, 10% acinar	N0	- negativo
		Bien diferenciado	M0	*Genes mutados:*
		STAS: no se observa	I A2	*- EGFR* deleción en el exón 19
		Sin invasión de pleura visceral (PL0)		*Genes no mutados:*
		Sin invasión linfovascular		*- ALK, ROS1, KRAS, NRAS, BRAF*
LSI	20 mm	Adenocarcinoma invasivo	pT1b	*PD-L1:*
		55% lepídico, 40% acinar, 5% papilar	N0	- negativo
		Bien diferenciado	M0	*Genes no mutados:*
		STAS: presente	I A2	*- EGFR, ALK, ROS1, KRAS, NRAS, BRAF*
		Sin invasión de pleura visceral (PL0)		
		Sin invasión linfovascular		

*: diámetro tumoral mayor; TNM: estadio tumoral atendiendo al tamaño (T), afectación ganglionar (N) y presencia de metástasis (M) según la *American Joint Committee on Cancer* (AJCC), 8ª edición; LSD: lóbulo superior derecho. LSI: lóbulo superior izquierdo; STAS: extensión tumoral a través de espacios aéreos; PD-L1: análisis mediante inmunohistoquímica. *EGFR*, *KRAS*, *NRAS* y *BRAF*: análisis inicial mediante PCR y, posteriormente, incluidos en la secuenciación de nueva generación (NGS); *ALK* y *ROS1*: análisis incluido en la NGS (en LSD_2_ y LSI se realizó inicialmente el análisis mediante inmunohistoquímica).

## DISCUSIÓN

Dado el incremento del uso de la TC en la detección del cáncer de pulmón, se informan cada vez más casos de CPPM^2^^,^^4^. La incidencia del CPPM sincrónico varía entre el 0,2% y el 6,2%^5^. Al menos la mitad de estos pacientes tienen tumores con histología idéntica, y en más de dos tercios de estos casos las lesiones se encuentran en el pulmón ipsilateral^6^.

Con el aumento de la esperanza de vida, algunas personas con cáncer de pulmón desarrollan un segundo cáncer de pulmón primario (CPPM metacrónico), con una incidencia acumulada aproximada del 20% después de la resección quirúrgica, tanto entre las que nunca fumaron como entre las que alguna vez fumaron^2^.

Beyreuther describió en 1924 el primer caso de CPPM^5^ y en 1975 Martini y Melamed propusieron los primeros criterios diagnósticos^7^, ampliamente usados durante años. La Asociación Internacional para el Estudio del Cáncer de Pulmón (IASLC) elaboró en 2016 una propuesta de clasificación del cáncer de pulmón con afectación pulmonar múltiple en cuatro tipos: 1) segundo cáncer de pulmón primario (CPPM sincrónico), 2) cáncer de pulmón primario con nódulos tumorales separados que tienen la misma histología (metástasis intrapulmonar), 3) cáncer de pulmón multifocal en vidrio deslustrado/lepídico, y 4) cáncer de pulmón de tipo neumónico difuso^1^.

El tipo y la morfología radiológica en la TC de los nódulos pulmonares puede ayudar a diferenciar entre un CPPM y una metástasis intrapulmonar. Cuando se detectan dos nódulos pulmonares sospechosos de malignidad y ambas lesiones son parcialmente sólidas (predominantemente sólidas) o nódulos sólidos puros, y no muestran bordes espiculados ni broncograma aéreo en la TC, se debe considerar con alta probabilidad la presencia de una metástasis intrapulmonar^8^. En este contexto, la paciente tenía tres lesiones pulmonares: una masa solida pulmonar en el LSD de bordes espiculados (LSD_1_), un nódulo parcialmente sólido en el LSD con signo de broncograma aéreo (LSD_2_) y otro nódulo en vidrio deslustrado en el LSI; siendo este último más sugestivo de CPPM sincrónico que de metástasis intrapulmonar contralateral.

En ocasiones, y a pesar de los criterios establecidos, sigue siendo un desafío distinguir entre CPPM y metástasis intrapulmonar^2^. Sin embargo, es crucial diferenciarlos ya que si, por ejemplo, el CPPM se diagnostica erróneamente como metástasis intrapulmonar, el paciente perdería la oportunidad de tratamiento quirúrgico o podría presentar efectos secundarios causados por dosis innecesarias de quimioterapia y/o radioterapia. Por el contrario, si la metástasis intrapulmonar se diagnostica como CPPM, la supervivencia global del paciente se vería negativamente afectada^4^.

El CPPM sincrónico y el cáncer de pulmón multifocal en vidrio deslustrado/lepídico se consideran lesiones primarias múltiples que se originan de forma independiente y tienen diferentes comportamientos biológicos y pronósticos^9^. Sin embargo, un metaanálisis publicado en el 2020^9^ describió que la mayoría de los estudios previamente publicados, y a efectos de evaluar la supervivencia global y los factores pronósticos, consideraban estos dos patrones de enfermedad como CPPM sincrónicos, lo que puede conducir a discrepancias respecto a los resultados quirúrgicos reales.

En cuanto al manejo, la resección quirúrgica sigue siendo el principal tratamiento para el CPPM. Tanto la lobectomía como la resección sublobar (segmentectomía anatómica o resección atípica) son aceptables^2^^,^^4^, pero debe evitarse la neumonectomía^4^. La resección sublobar no parece afectar negativamente la supervivencia y es una alternativa razonable en algunos casos. No obstante, el tipo de resección debe ser evaluado por un equipo multidisciplinar experimentado^2^^,^^4^^,^^9^^,^^10^. En nuestro caso, en un primer momento se realizó una lobectomía pulmonar del LSD, seguida de una trisegmentectomía apical del LSI (resección anatómica del culmen); ambas intervenciones fueron abordadas mediante VATS biportal, sin complicaciones intraoperatorias.

Uno de los múltiples desafíos que pueden surgir al detectar un cáncer de pulmón con afectación pulmonar múltiple, es cuando el tumor secundario es del mismo tipo histológico que el primario. En estas circunstancias, y más cuando, como en nuestro caso, son triples tumores sincrónicos, es difícil afirmar que los tumores sean de orígenes independientes en el pulmón, ya que es posible que el tumor secundario sea una extensión del tumor original^11^.

Aunque las características histológicas son útiles, a menudo es necesario un análisis molecular, como la NGS realizada en nuestro caso, que contribuya a distinguir el CPPM sincrónico de las metástasis intrapulmonares^2^. Eguren-Santamaria y col^12^ realizaron en 2018 un panel de secuenciación de ADN dirigida de 50 genes en un paciente con tres tumores de pulmón resecados, y sus resultados respaldaron el diagnóstico de CPPM frente al de un único cáncer de pulmón avanzado. Por tanto, la secuenciación de ADN dirigida aumenta significativamente la precisión diagnóstica en pacientes con múltiples tumores de pulmón.

En la literatura se encuentran algunos estudios que han incluido casos de pacientes con tres o más tumores pulmonares sincrónicos^5^^,^^10^. También se han publicado casos aislados de, por ejemplo, triples tumores pulmonares primarios sincrónicos histológicamente distintos, en diferente lóbulo^11^ o en el mismo lóbulo^13^. Sin embargo, la identificación del mismo tipo histológico con afectación pulmonar bilateral en nuestro caso es un hallazgo poco frecuente.

Se ha presentado un caso de triple carcinoma pulmonar primario sincrónico cuyas lesiones eran del mismo tipo histológico pero presentaban diferentes patrones histológicos y características moleculares, con el objetivo de destacar la importancia tanto de realizar un estudio anatomopatológico detallado para obtener un diagnóstico preciso como de que estos pacientes con cáncer de pulmón con afectación pulmonar múltiple sean manejados por un grupo multidisciplinar experimentado.

## Data Availability

Se encuentran disponibles bajo petición al autor de correspondencia.
